# The Year

**Published:** 1897-01

**Authors:** 


A Monthly Review of Medicine and Surgery.

*EDITORS:*

THOMAS LOTHROP, M. D. - - WM. WARREN POTTER, M. D.

All communications, whether of a literary or business nature, should be addressed to the managing editor: 284 FRANKLIN STREET, BUFFALO, N. Y.

VOL. XXXVI. JANUARY, 1897. No. 6.

### THE YEAR.

THOUGH the year just closed has not been fraught with great achievements or startling developments in so far as relates to the local medical profession, yet there have been some incidents that deserve to be remembered, as well as some good work done, and we appropriate a little place to a review of such as seem most important.

One of the most interesting events of the year was the observance of its 75th anniversary by the medical society of the county of Erie at its regular annual meeting, held January 14, 1896. The attendance was large, and great interest was manifested in the proceedings. Dr. Franklin C. Gram, secretary, read a historical sketch of the society, beginning with its organisation in 1821 and bringing it forward to the present time. It was full of incidents, interspersed with humorous illusions, and is a decided contribution to the archives of the society.

Dr. John Hauenstein, who became a member of the society in 1844, read a paper giving some reminiscences of the profession in its earlier days, and told of the first uses of anesthetics in the county. Dr. C. C. Wyckoff read a paper giving an account of the establishment of Buffalo University Medical College, and Dr. John Cronyn contributed a similar one relating to Niagara University Medical College. Dr. Edward Storck presented a paper relating to the work of the board of censors during the twelve years he was chairman that possessed historical value. All these papers have been printed in the JOURNAL; likewise a separate reprint of them has been issued to the members of the society.

The Buffalo Academy of Medicine has held its regular quarterly meetings, and the various sections thereof their usual weekly sessions, on which occasions interesting papers have been read and discussed and clinical cases reported. If the academy had published a record of its work it would be convenient to present a synopsis or an analysis of it at this time. We desire to accentuate the importance of publishing an accurate record of society work in the local journal, for whoever writes the history of the profession in the future will sadly miss such a record of the academy's proceedings during the last four years. The medical profession of a city like Buffalo should find itself recorded in the pages of its local journal, both in the contribution of papers and in accurate society reports. Papers published in journals away from home do little permanent good to authors, because they are scattered here and there and are not accessible to either historian or the compiler of home scientific work.

In several instances prominent men from a distance have contributed papers during the year, among whom we may mention Dr. A. C. Abbott and Dr. John B. Deaver, of Philadelphia, and Dr. C. B. Nancrede, of Ann Arbor. This plan is to be commended, and we hope will be repeated at frequent intervals. It gives a freshness to the character of the meetings and serves to stimulate enthusiasm. It ought to be remembered, however, that the academy owes it to its guests on such occasions to give them full houses.

The private medical societies, among which we may mention the Medical Club, the Medical Union, the Roswell Park Medical Club and the Buffalo Microscopical Society, are also to be credited with the usual amount of good work, but again records are inaccessible.

Mr. George W. Rafter, civil engineer, of Rochester, read a paper before the Microscopical Club on Lake Erie as a Water Supply, which was published in this journal.

The JOURNAL surprised and probably startled some of its readers and friends by publishing a Woman's Edition in June. This issue, in all its departments, was the work of women physicians, and it may be truthfully asserted that it was quite equal in quality and appearance to its other eleven numbers. At all events, it was commended for its excellence by its contemporaries and readers.

One of the principal events of interest during the year was the meeting of the American Public Health Association, held September 15-18, 1896. This distinguished body came to Buffalo in large numbers and held one of its most interesting and successful meetings. It was presided over by Dr. Eduardo Licéaga, a distinguished physician from Mexico, who brought with him a delegation of thirty members. A synopsis of its proceedings appeared in this journal for October, 1896. Among the officers which it elected for the current year was Dr. Ernest Wende, vice-president, which was a fitting recognition of his excellent work as chairman of the local executive committee, and especially for his splendid record as health commissioner of Buffalo.

The semicentennial anniversary of the introduction of surgical anesthesia was commemorated at Buffalo University Medical College, October 16, 1896, with public exercises. Introductory remarks were made by the Hon. Jas. O. Putnam, chancellor; a historic address was made by Prof. Roswell Park, and some interesting reminiscences were related by Dr. E. M. Moore, of Rochester. Dr. Hauenstein and Dr. Wyckoff, of this city, also added a few items of local interest.

The literary contribution of the year is a system of surgery by American authors, edited by Dr. Roswell Park, of Buffalo, who is likewise the principal author of the work.

It will readily be observed from this synopsis that, taken altogether, while perhaps, as we have before remarked, no startling achievements have been made by the medical profession in this vicinity during the year 1896, yet the record is an honorable one and will result in the accomplishment of much good. A subject for special congratulation is that we have been singularly exempt from sickness and death in our ranks, a blessing for which we are all truly thankful. As a final remark, let us hope that the coming year, 1897, will yield as much of happiness and professional credit as did its predecessor.

